# Synergistic or Additive Pharmacological Interactions between Magnoflorine and Cisplatin in Human Cancer Cells of Different Histological Origin

**DOI:** 10.3390/ijms21082848

**Published:** 2020-04-19

**Authors:** Estera Okon, Jarogniew J. Luszczki, Wirginia Kukula-Koch, Marta Halasa, Agata Jarzab, Daariimaa Khurelbat, Andrzej Stepulak, Anna Wawruszak

**Affiliations:** 1 Department of Biochemistry and Molecular Biology, Medical University of Lublin, Chodzki 1 St., 20-093 Lublin, Poland; estera.okon@umlub.pl (E.O.); martahalasa@umlub.pl (M.H.); agatajarzab@umlub.pl (A.J.); andrzejstepulak@umlub.pl (A.S.); 2 Department of Pathophysiology, Medical University of Lublin, Jaczewskiego 8b St., 20-081 Lublin, Poland; jarogniew.luszczki@umlub.pl; 3 Department of Pharmacognosy, Medical University of Lublin, Chodzki 1 St., 20-093 Lublin, Poland; virginia.kukula@gmail.com; 4 Department of Pharmaceutical Chemistry and Pharmacognosy, School of Pharmacy, Mongolian National University of Medical Sciences, Zorig St., Ulaanbaatar 14210, Mongolia; daariimaa@mnums.edu.mn

**Keywords:** magnoflorine, cisplatin, isobolographic analysis, breast cancer, lung cancer, glioblastoma, rhabdomyosarcoma

## Abstract

Magnoflorine is an aporphine alkaloid present in plant species belonging to the Berberidaceae, Magnoliaceae, Menispermaceae, or Papaveraceae botanical families. The interest of magnoflorine has increased recently due to its multiplicity of pharmacological properties. The aim of this study was the analysis of combined anti-proliferative effect of magnoflorine and cisplatin and the assessment of drug–drug pharmacological interaction between these agents using isobolographic method in MDA-MB-468 human breast, NCIH1299 lung, TE671 rhabdomyosarcoma, or T98G glioblastoma cancer cell lines. Magnoflorine in combination with cisplatin at a fixed ratio of 1:1 augmented their anticancer action and yielded synergistic or additive pharmacological interactions by means of isobolographic method, therefore combined therapy using these two active agents can be a promising chemotherapy regimen in the treatment of some types of breast, lung, rhabdomyosarcoma, and glioblastoma cancers.

## 1. Introduction

Cis-diamminedichloroplatinum (II) (cisplatin, CDDP), due to its broad spectrum of clinical activity and high efficacy, is one of the most extensively used chemotherapeutic drug and anticancer agent for treatment of numerous solid tumors [1,2,3], including testicular cancer [4], ovarian cancer [5], breast cancer [6], cervical cancer [7], non-small cell lung cancer [8], head and neck cancer [9], bladder cancer [10] and others [1,3,11]. Anticancer activity of CDDP has also been demonstrated when it is used in combination with other types of anticancer therapy, including radiotherapy [12], chemotherapy [13] or immunotherapy [3,14]. Unfortunately, the clinical use of CDDP has been largely limited due to its serious severe side effects including nephrotoxicity [15,16], ototoxicity [15], neurotoxicity [17], cardiotoxicity [18], gastrointestinal toxicity [19], hepatotoxicity [16], hematological toxicity [20]. All these side effects require lowering the dosage or even stopping administration of the drug [1]. 

It has been shown that compounds of natural origin can be effective in the treatment of cancer, either in monotherapy or combination therapy [21]. Magnoflorine (MGN) could be proposed as a promising natural active agent for potent application in cancer therapy [22]. MGN is a plant-derived quaternary isoquinoline alkaloid widely distributed within the representatives of several botanical families, such as Magnoliaceae, Berberidaceae, Menispermaceae or Papaveraceae [23]. MGN has been the subject of intensive investigations on account of its multiple diverse biochemical and pharmacological activities including anti-inflammatory [24], immunomodulatory [25], neuropsychopharmacological [26], anti-anxiety [24,27], antioxidant [28], antiviral [28] or antifungal [29] activities. It has been demonstrated that oral administration of MGN significantly decreased the fasting serum glucose and inhibited the up-regulation of blood glucose level [30]. MGN has been reported to reduce arterial blood pressure and induced hypothermia in in vivo models. It was also found to possess anti-HIV activity [23]. A few studies demonstrated the anticancer activity of MGN. However, the mechanism of action as well as the effect of MGN on tumor progression remain unclear [22,31]. Interestingly, toxicity studies have suggested that MGN is non-toxic to most normal cells. Unfortunately, pharmacokinetic studies have revealed that MGN has a low bioavailability index and high absorption and elimination rates [24].

Therefore, in our study we assessed a combined anti-proliferative effect of MGN isolated from natural sources by countercurrent chromatography, applied together with CDDP in an experimental treatment against various types of cancer cells in order to determine their pharmacological interaction by means of advanced isobolographic analysis. 

## 2. Results

### 2.1. The Recovery of Magnoflorine from Plant Matrix

**Figure 1:**
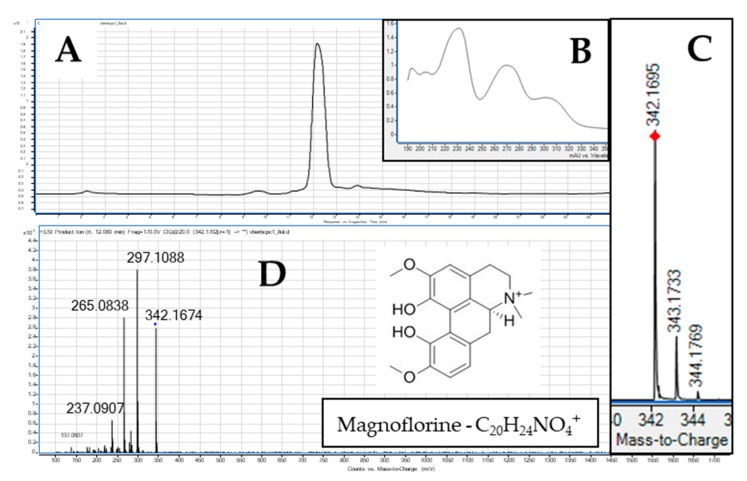
The purity of the isolated magnoflorine (**A**) presented in the mass chromatogram, its UV spectrum (**B**), the isotopic distribution of the parent ion (**C**), and the fragmentation spectrum (**D**) obtained at the collision energy of 20 V in the HPLC-MS analysis.

The hydrostatic countercurrent chromatography (also known as centrifugal partition chromatography—CPC) operated under optimized conditions was found selective enough to purify MGN from the overground parts of Siberian barberry (*Berberis sibirica* Pall.) that are rich sources of both phenolic compounds and alkaloids [32]. The addition of an acid and a base to the separation system helped to obtain this aporphine alkaloid directly from the plant matrix, separately from abundantly present phenolic compounds, in the pH-zone refining mode of separation. As with the previous report [32], MGN was eluted in the 5th fraction and its purity and identity checked in an HPLC-MS (High Performance Liquid Chromatography coupled with Mass Spectrometry) experiment prior to the in vitro tests on cell lines (Figure 1). Each injection of 1 g of the total extract provided ca. 25 mg of high purity MGN for further studies. 

The identification of MGN in the fraction was based on the accurate mass measurements, the UV spectrum, the isotopic distribution of the parent ion, and the study of its fragmentation pattern. The obtained results were consistent with the scientific literature and the available libraries of mass spectra (METLIN). The MS chromatograms recorded in the positive ionization mode show clear *m*/*z* signals that come from the detachment of methyl, ammonium, and hydroxyl functional groups, or carbon monoxide out of the parent ion [M^+^]. The *m*/*z* signal at 297 *m*/*z* confirms the loss of two methyl groups and NH group [M−NH-(CH_3_)_2_]^+^ at the 4° ammonium ion, a weak signal at 282—the loss of three methyl groups and one NH group and the *m*/*z* signal at 265 that confirms the detachment of additional –CH_3_OH group out of the *m*/*z* signal of 297 [33,34,35]. The *m*/*z* of 237 shows a subsequent loss of –CO group out of the 265. High resolution mass spectra determined the structure of MGN with high accuracy and low error of measurement equal to -0.63 ppm. The double bond equivalents number of the metabolite was determined as 10. This alkaloid is characterized by the following maxima in the UV spectrum: 231, 270, 305 (Figure 1B).

### 2.2. MGN and CDDP Administered Individually or in Combination Decrease Proliferation of TE671, T98G, MDA-MB-468, and NCIH1299 Cancer Cells

**Figure 2:**
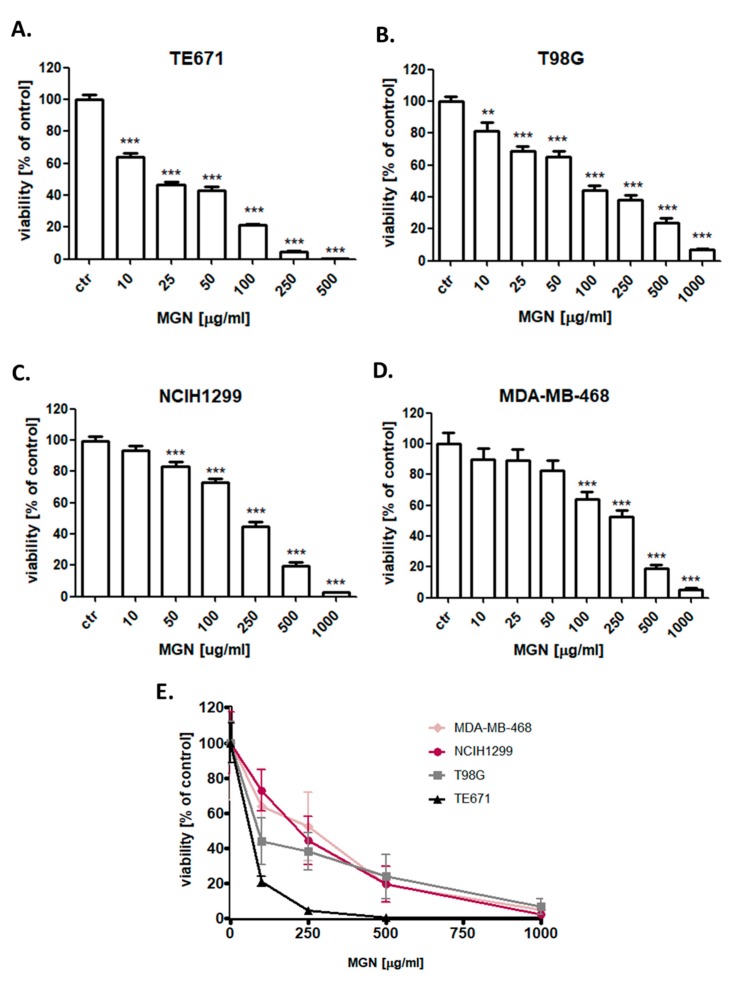
The anti-proliferative effects of magnoflorine (MGN) in (**A**) TE671 (**B**) T98G (**C**) NCIH1299 (**D**) MDA-MB-468 and (**E**) all analyzed cancer cell lines after 96 h treatment with various concentrations (10–1000 μg/mL) of an active agent. The cell viability was measured by the MTT assay. Results were analyzed using GraphPad Prism 5.0 software (one-way ANOVA; Tukey post-hoc testing). Statistical differences were considered relevant at *p* < 0.05 (** *p* < 0.01, *** *p* < 0.001). Data are expressed as mean ± standard deviation of the mean (± SD); *n* = 24 per concentration from three independent experiments.

**Figure 3:**
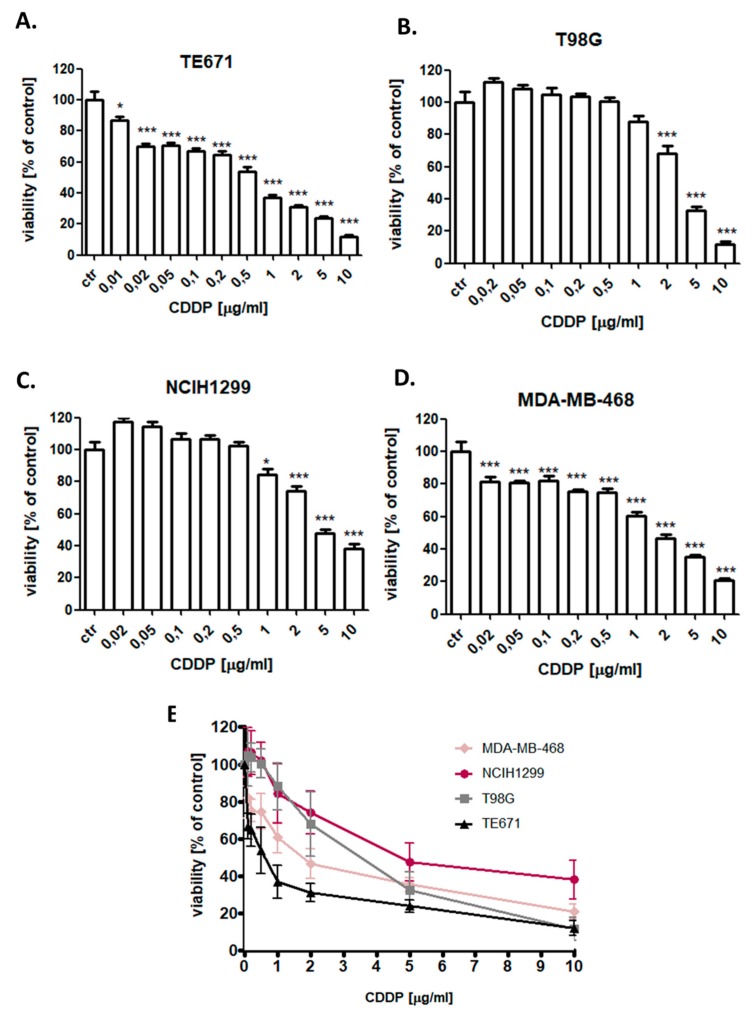
The anti-proliferative effects of cisplatin (CDDP) in (**A**) TE671 (**B**) T98G (**C**) NCIH1299 (**D**) MDA-MB-468 and (**E**) all analyzed cancer cell lines after 96 h treatment with various concentrations (0.01–10 μg/mL) of an active agent. The cell viability was measured by the MTT assay. Results were analyzed using GraphPad Prism 5.0 software (one-way ANOVA; Tukey post-hoc testing). Statistical differences were considered relevant at *p* < 0.05 (* *p* < 0.05, *** *p* < 0.001). Data are expressed as mean ± standard deviation of the mean (± SD); *n* = 24 per concentration from three independent experiments.

**Figure 4:**
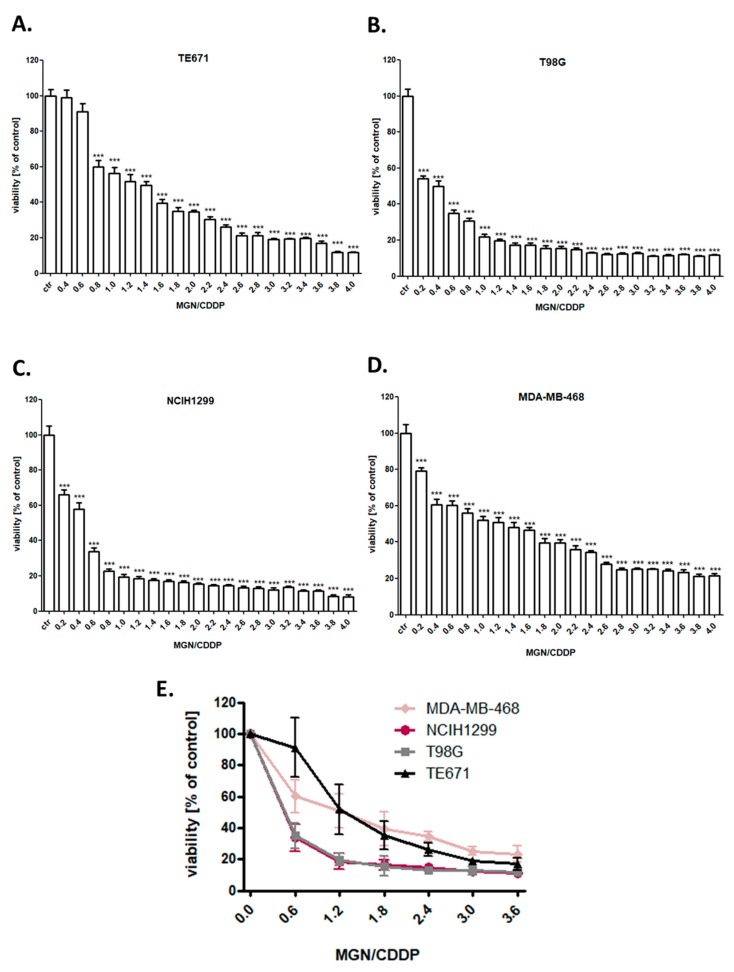
The anti-proliferative effects of combined treatment of magnoflorine (MGN) and cisplatin (CDDP) in (**A**) TE671 (**B**) T98G (**C**) NCIH1299 (**D**) MDA-MB-468 and (**E**) all analyzed cancer cell lines after 96 h treatment with 1:1 drug mixture (MGN/CDDP) in increasing concentrations. Cancer cells were exposed to concomitant MGN and CDDP treatment using different ratios of the IC_50_ (2.0 = IC_50_ + IC_50_). The cell viability was measured by the MTT assay. Results were analyzed using GraphPad Prism 5.0 software (one-way ANOVA; Tukey post-hoc testing). Statistical differences were considered relevant at *p* < 0.05 (*** *p* < 0.001). Data are expressed as mean ± standard deviation of the mean (± SD); *n* = 24 per concentration from three independent experiments.

The cytotoxic effect of MGN and CDDP was determined in the TE671, T98G, MDA-MB-468, and NCIH1299 cancer cell lines using the 3-(4,5-dimethylthiazol-2-yl)-2,5-diphenyltetrazolium bromide (MTT) assay in order to establish the IC_50_ value for each analyzed compound in all cell lines. IC_50_ values for all investigated cell lines were depicted in Table 1. All cancer cells were exposed to either culture medium (control) or increasing concentrations of MGN (10–1000 µg/mL) (Figure 2) or CDDP (0.01–10 µg/mL) (Figure 3) individually and in MGN/CDDP combination (Figure 4). In our study, we have demonstrated the dose-dependent growth inhibition effect of both compounds in all analyzed cancer cell lines. TE671 was the most sensitive cell line both to MGN (Figure 2) and CDDP (Figure 3) treatment individually. Interestingly, this cell line was the least sensitive to MGN/CDDP combined treatment (Figure 4). We observed that T98G and NCIH1299 cells were the most sensitive to MGN/CDDP treatments among all analyzed cancer cell lines (Figure 4).

### 2.3. Influence of MGN, CDDP and Their Combination (at the Fixed Drug Concentration Ratio of 1:1) on the Anti-Proliferative Activity of TE671, T98G, NCIH1299, and MDA-MB-468 Cancer Cells 

**Figure 5:**
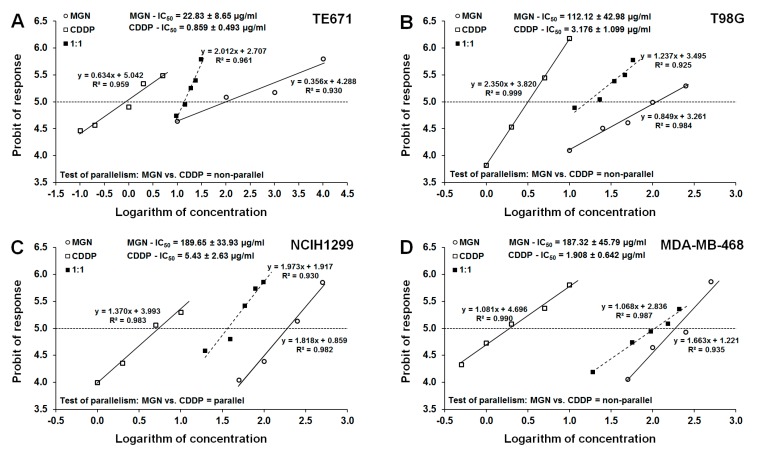
Log-probit analysis of concentration–response relationship lines for MGN and CDDP in TE671, T98G, NCIH1299, and MDA-MB-468 cancer cell lines. (**A**) Log-probit analysis of concentration–response relationship lines for MGN and CDDP in the TE671 cancer cell line. (**B**) Log-probit analysis of concentration–response relationship lines for MGN and CDDP in the T98G cancer cell line. (**C**) Log-probit analysis of concentration–response relationship lines for MGN and CDDP in the NCIH1299 cancer cell line. (**D**) Log-probit analysis of concentration–response relationship lines for MGN and CDDP in the MDA-MB-468 cancer cell line. Concentrations of MGN and CDDP, when administered singly and in combination at the fixed drug concentration ratio of 1:1, were transformed into logarithms to the base 10, while the anti-proliferative activity of the studied drugs in four cancer cell lines (TE671, T98G, NCIH1299, and MDA-MB-468) were transformed into probits according to Litchfield and Wilcoxon (1949). Each point on each graph illustrates the experimentally derived concentration-effect for the respective drugs (MGN and CDDP) and their mixture at the fixed ratio of 1:1. For each drug and mixture tested, at least 4-5 points create a line reflecting the concentration-effect, whose equation is presented close to the analyzed line on each graph. The dotted line on each graph, which is parallel to X axis and starts from 5 probit, illustrates a 50% anti-proliferative effect for the investigated MGN, CDDP, and their mixture at the fixed ratio of 1:1. This dotted line, by crossing the concentration-effect lines for the drugs and their mixtures, determines in approximation their logarithms of IC_50_ values. This multipart figure contains equations of concentration–response relationship lines for MGN and CDDP along with their IC_50_ values, when administered alone. Results from the test of parallelism are placed above the X axis on each graph. This test allows for direct comparison of concentration–response relationship lines for MGN and CDDP (when administered alone) with respect to their mutual parallelism. If MGN and CDDP had their concentration-effect lines nonparallel, the type I isobolographic analysis for nonparallel concentration-effect lines was used. Otherwise, the interaction was analyzed with type I isobolographic analysis for collateral concentration–response relationship lines. At the top of each graph, the IC_50_ values (± S.E.M.) for MGN and CDDP are presented.

Both MGN and CDDP produced clearly defined anti-proliferative activity in four tested cancer cell lines. Log-probit analysis of concentration–response relationship effects between MGN and CDDP allowed calculating the median inhibitory concentrations (IC_50_ values ± S.E.M.) for MGN and CDDP in TE671, T98G, NCIH1299, and MDA-MB-468 cancer cell lines (Table 1). Test of parallelism examining concentration–response relationship lines for MGN and CDDP revealed that MGN had its concentration–response line collateral to that of CDDP only in the NCIH1299 cancer cell line (Table 1, Figure 5C). On the contrary, MGN had its concentration–response relationship line nonparallel to that of CDDP in the TE671, T98G, and MDA-MB-468 cancer cell lines (Table 1, Figure 5A,B,D).

### 2.4. Type I Isobolographic Analysis for Parallel and Nonparallel Concentration–Response Relationship Lines for the Combination of MGN and CDDP (at the Fixed Ratio of 1:1) in the TE671, T98G, NCIH1299, and MDA-MB-468 Cancer Cell Lines

**Figure 6:**
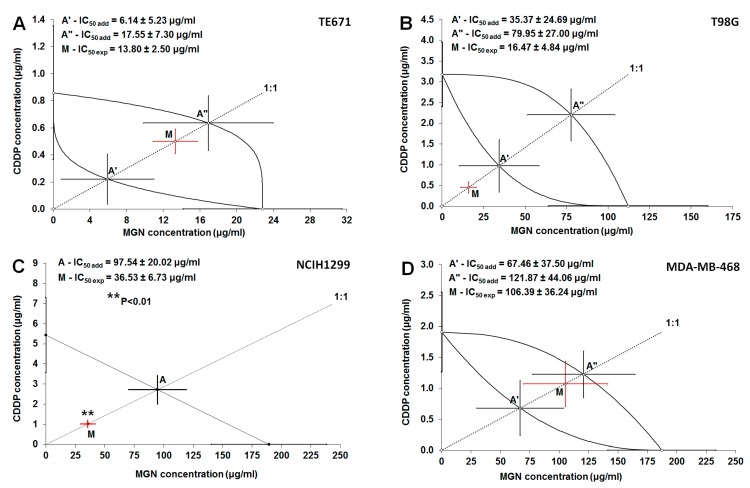
Isobolograms for additive and synergistic interactions between MGN and CDDP in four cancer cell lines. (**A**) Isobologram for the additive interaction of MGN with CDDP in the TE671 cancer cell line. (**B**) Isobologram for the additive interaction of MGN with CDDP in the T98G cancer cell line. (**C**) Isobologram for the synergistic interaction of MGN with CDDP in the NCIH1299 cancer cell line; (**D**) Isobologram for the additive interaction of MGN with CDDP in the MDA-MB-468 cancer cell line. The IC_50_ ± S.E.M. for MGN and CDDP are plotted on the abscissa and ordinate of the Cartesian system of coordinates. The dotted line starting from the beginning of the Cartesian plot system illustrates the fixed drug concentration ratio of 1:1 for the mixture of two studied drugs (MGN and CDDP). The hyperbolic curves that connect the IC_50_ values placed on X- and Y-axes illustrate the lower and upper lines of additivity for nonparallel concentration-effects (a,b,d). For nonparallel concentration-effect relationship lines of MGN and CDDP (a,b,d), the lower and upper isoboles of additivity form the additivity area between the curves connecting the IC_50_ values for MGN and CDDP administered singly. Intersections of the dotted line for the fixed ratio of 1:1 with lower and upper lines of additivity illustrate, in approximation, the additivity points A’ and A”(a,b,d). The theoretically calculated IC_50 add_ values (± S.E.M.) are placed on the lower and upper isoboles of additivity as points A’ and A”, respectively. The experimentally derived IC_50 exp_ value (± S.E.M.) for the two-drug mixture of MGN and CDDP is placed as the point M, on the dotted line for the fixed ratio of 1:1. Since the point M is placed between the points A’ and A” within the area of additivity (a,d), the interaction between MGN and CDDP is additive in the TE671 and MDA-MB-468 cancer cell lines. Since the point M is placed close to the point A’ for the lower isobole of additivity (b), the interaction is additive with a tendency towards synergy in the T98G cancer cell line. For parallel concentration-effect relationship lines of MGN and CDDP (c), the straight diagonal line of additivity connects the IC_50_ values placed on X- and Y-axes for MGN and CDDP administered singly. Intersection of the dotted line for the fixed ratio of 1:1 with the line of additivity illustrates, in approximation, the point of additivity A (c). The theoretically calculated IC_50 add_ value (± S.E.M.) is placed on the line of additivity as the point A. The experimentally derived IC_50 exp_ value (± S.E.M.) for the two-drug mixture of MGN and CDDP is placed on the graph as the point M. Since the point M is placed significantly below the point A (c), the interaction between MGN and CDDP is supra-additive (synergistic) in the NCIH1299 cancer cell line. ** *p* < 0.01 vs. IC_50_ add.

The combination of MGN with CDDP at the fixed drug concentration ratio of 1:1 exerted a clear-cut anti-proliferative activity on four investigated (TE671, T98G, NCIH1299, and MDA-MB-468) cancer cell lines (Table 2; Figure 6A–D). 

The experimentally derived IC_50 exp_ value for the mixture of MGN with CDDP in the cancer TE671 cell line was 13.80 ± 2.50 μg/mL (Table 2; Figure 6A). The type I isobolographic analysis for nonparallel concentration–response relationship lines did not reveal any significant differences between the IC_50 exp_ and IC_50 add_ values with unpaired Student’s t-test with Welch’s correction in the cancer TE671 cell line (Table 2; Figure 6A).

The IC_50 exp_ value for the mixture of MGN with CDDP in the T98G cancer cell line was 16.47 ± 4.84 μg/mL (Table 2; Figure 6B). With the type I isobolographic analysis for nonparallel concentration–response relationship lines, no significant differences were found between the IC_50 exp_ and IC_50 add_ values (with unpaired Student’s t-test with Welch’s correction) in the T98G cancer cell line (Table 2; Figure 6B).

In contrast, the IC_50 exp_ value for the mixture of MGN with CDDP in the NCIH1299 cancer cell line was 36.53 ± 6.73 μg/mL and considerably differed (*p* < 0.01; unpaired Student’s t-test with Welch’s correction) from that for IC_50 add_ value, which amounted to 97.54 ± 20.02 μg/mL (Table 2; Figure 6C). With the type I isobolographic analysis for parallel concentration–response relationship lines, the interaction between MGN and CDDP was supra-additive (synergistic) in the NCIH1299 cancer cell line (Table 2; Figure 6C).

The IC_50 exp_ value for the last mixture of MGN with CDDP in the MDA-MB-468 cancer cell line was 106.39 ± 36.24 μg/mL (Table 2; Figure 6D). The type I isobolographic analysis for nonparallel concentration–response relationship lines revealed that the IC_50 exp_ and IC_50 add_ values did not differ significantly (with unpaired Student’s t-test with Welch’s correction) in the MDA-MB-468 cancer cell line (Table 2; Figure 6D).

### 2.5. Isobolographic Analysis of the Types of Pharmacodynamic Interaction Between MGN and CDDP (at the Fixed Drug Concentration Ratio of 1:1) in the TE671, T98G, NCIH1299, and MDA-MB-468 Cancer Cell Lines

Isobolographic analysis of interaction for nonparallel concentration–response relationship lines revealed that the mixture of MGN with CDDP at the fixed ratio of 1:1 exerted additive interaction in the TE671 and MDA-MB-468 cancer cell lines. Similarly, the mixture of MGN with CDDP in the T98G cancer cell line exerted additivity with a slight tendency towards synergy. In contrast, synergistic interaction was isobolographically observed for the mixture of MGN with CDDP in the NCIH1299 cancer cell line. Current study indicates that MGN may be used together with CDDP as a two-drug therapy against NCIH1299 cancer. Summing up, this study revealed that MGN may become in future a potential therapeutic agent in the therapy with CDDP against some specific cancers.

## 3. Discussion

Despite significant progress in the development of novel therapeutic options, chemotherapy of cancers still does not bring expected results [39,40,41]. Therefore, combinations of established anticancer chemotherapeutics and new natural active agents are being tested in order to improve clinical outcomes of oncological patients [42,43,44]. It has been shown that bioactive components from plants and other natural sources can not only reduce the effective doses of chemotherapeutic drugs but also sensitize cancer cells to standard cytotoxic treatment, strengthen the combined therapeutic activity of both active agents and limit cytotoxic effect in relation to normal cells. Moreover, combined therapy with natural active agents is able to diminish MDR of chemotherapeutic drugs [45]. Lowering the therapeutic doses of standard cytostatics by partially replacing them with natural herbal medicines can significantly reduce the cytotoxicity of currently used chemotherapeutic methods [43,46]. 

CDDP is an anticancer drug which is widely used for the treatment of various solid malignancies [47]. However, chemotherapy using CDDP is limited due to the low therapeutic index, several undesirable adverse effects, high toxicity relative to the normal cells, as well as the occurrence of drug resistance. Combined therapy with the use of two or more drugs with different mechanisms of action is often used to overcome these problems [39,48,49]. 

It has been shown that many substances of natural origin are well tolerated by oncological patients and do not cause serious side effects [50,51,52]. New natural active agents which effectively and selectively eliminate carcinoma cells, and additionally augment anticancer activities of currently used chemotherapeutic drugs without demolishing healthy tissue, are of great importance. MGN seems to meet these criteria. It has been reported that MGN shows anticancer activity against several cancer cell types in vitro [53]. It has also been reported that MGN has much lower toxicity on normal cells, in contrast to cancer cells. However, the mechanism of action of MGN has not been fully understood yet [22].

In the present study we propose a combination of CDDP-standard chemotherapeutic agent with a MGN-natural compound that do not strongly affect human normal cells [22]. The results obtained in our experiments show that both CDDP and MGN significantly reduce cell viability of all tested cancer cells. MGN and CDDP were evaluated for their anticancer activity against human breast, lung, rhabdomyosarcoma, and glioblastoma cancer cell lines and exhibited dose-dependent growth inhibition. In the present study, we also show that MGN enhance the cytotoxicity of CDDP in all analyzed cancer cell lines. As with our results, it has been reported that MGN strongly reduced viability of MCF7, MDA-MB-231, MDA-MB-453, and BT474 breast cancer cells without affecting MCF-10A human normal mammary epithelial cell line [21,22]. Moreover, MGN obtained from fractionation of the methanol extract from *Magnolia grandiflora* leaves inhibited cell viability of the U251 brain tumor cell line, Hela cervix tumor cell line and HEPG2 hepatocellular carcinoma cell line. Interestingly, IC_50_ of MGN against HEPG2 cells was only two times higher than IC_50_ of doxorubicin (DOX) [21,53]. Additionally, it has been reported that aqueous extract from *Coptidis rhizoma* (CRAE) containing 2.2% of MGN exerted 50% of cytotoxicity against HepG2 and MHCC97L (cellosaurus cell line) cancer cells after 48 h at the doses of 120 μg/mL and 150 μg/mL, respectively. Furthermore, CRAE treatment increased the phosphorylation of eukaryotic elongation factor 2 (eEF2) and inhibited vascular endothelial growth factor (VEGF) synthesis in HepG2 and MHCC97L cells. There has also been observed a reduction of tumor size and neovascularization level in vivo in mice xenograft model after CRAE treatment. The presence of CD31-postive cells also decreased in CRAE-treated mice, demonstrating the decreased rate of blood vessel formation. Additionally, CRAE-treated mice showed a lower vascular density in cancer compared to the control group [54]. Other authors revealed that *Trichospora cordifolia* extract shows cytotoxicity against KB human oral squamous carcinoma and CHOK-1 hamster ovary cells with IC_50_ value of 52.7 μg/mL and 18.5 μg/mL, respectively [21,31].

In our studies, concomitant administration of CDDP and MGN allowed to reduce the doses of CDDP while achieving a similar or better anti-proliferative effect. In the present study, we analyzed efficacy of treatment and the type of pharmacological drug–drug interaction between MGN and CDDP to assess potential application of combined treatment in some types of cancer cells. On the basis of the isobolographic method that allows the precise characterization of drug–drug interaction, we have shown that the mixture of CDDP with MGN at the fixed-ratio of 1:1 exerted an additive or supra-additive (synergistic) pharmacological interactions in TE671 human rhabdomyosarcoma, T98G human glioblastoma, MDA-MB-468 human breast cancer and NCIH1299 non-small lung cancer cells, resulting in a greatly enhanced anticancer effect of these drugs’ combination compared to single drug treatment. No antagonism between tested substances was observed. Therefore, combined therapy using these two active agents can be a promising chemotherapy regimen in the treatment of some types of cancers. Beneficial effects of MGN/CDDP treatment observed in many types of cancer cells in vitro show that this combination could be regarded as a general phenomenon and occurs regardless of the histological origin of cancer cells. In our previous studies, we demonstrated synergistic or additive interaction between CDDP and other natural active agents such as curcumin [55] or osthole [56]. We have also shown the beneficial effect of combined CDDP and histone deacetylase inhibitors treatment against T47D, MCF7, MDA-MB-231 breast cancer [39,57], A549, NCI-H1563 human adenocarcinoma, NCI-H2170 human squamous cell carcinoma [49], TE671 human rhabdomyosarcoma [58] and RK33 human larynx cancer cells [59], using advanced isobolographic analysis of drug–drug interaction. The isobolography is the best, very rigorous and precise pharmacodynamic method to characterize and establish the type of interaction between active agents which exhibit a broad range of concentrations in both in vitro and in vivo studies. However, this very efficient method is not commonly used to determine the types of pharmacological drug–drug interactions in cancer related studies. Instead, simple correlations between tested agents are usually demonstrated, where only one or a few random chosen doses are selected [39,49]. So far, no studies assessing the impact of MGN and CDDP have been published.

A synergistic action of MGN and another chemotherapeutic drug—DOX in MCF7, MDA-MB-231, MDA-MB-453, and BT474 breast cancer cells—has previously been reported. MGN/DOX treatment significantly inhibited proliferation, migration, and invasion of breast cancer cells, as well as induced apoptosis through mitochondria-dependent pathway and cell distribution in G2/M phases. Additionally, MGN/DOX treatment resulted in the activation of autophagy via LC3-II regulated through p38 and phosphatidylinositol-3-kinase/protein kinase B (PI3K/PKB) signaling pathways in breast cancer cells. MGN/DOX co-treatment also increased the expression of epithelial marker (E-cadherin) and decreased expression of mesenchymal (N-cadherin, vimentin) markers compared to DOX separately. Moreover, DOX reduced the expression of cyclin-dependent kinase 1 (CDK1), cyclin B1, and cyclin-dependent kinase 2 (CDK2), which was further decreased after DOX/MGN co-treatment. p62 expression was reduced, as well as p53 and p21 protein levels were induced after MGN/DOX treatment, compared to DOX individually. Interestingly, DOX/MGN showed also antitumor effect in MCF7 xenograft model with relatively low toxicity to kidney, liver, heart, or spleen. Western blotting analysis also demonstrated that expression of p53, cleaved caspase-3, LC3-II and phospho-p38 (p-p38) was induced, and phospho-mTOR (p-mTOR) and phospho-AKT (p-AKT) expression was reduced after DOX/MGN combinational treatment [21,22].

In conclusion, our study which used the isobolographic method of pharmacological drug–drug interaction analysis demonstrates that the combination of MGN with CDDP could be used in some types of cancer cells to improve their antitumor effects and decrease their doses compared to those administered separately. Concomitant use of MGN with CDDP could primarily overcome CDDP-associated resistance in cancer patients as well as decrease the doses of CDDP to minimize the side effects of this chemotherapeutic agent. Given that CDDP induces serious side effects, the use of less toxic doses of this conventional chemotherapeutic in combination with clinically available doses of MGN seems to be an interesting therapeutic alternative. Concurrent administration of both active agents may be a novel strategy to enhance the efficacy of currently used anticancer therapies in cancer patients and more successfully eliminate carcinoma cells. By showing synergistic or additive interaction of tested compounds in analyzed cancer cells, our results strongly suggest application of this drug combination in other pre-clinical models, including animal xenografts.

## 4. Future Clinical Perspective

Lung cancer is the leading cause of cancer death worldwide. Platinum-based chemotherapy is still the first-line therapy for advanced non-small cell cancer [60]. Glioblastoma multiforme makes up more than 30% of all primary brain tumors. Despite the employment of multimodal anticancer treatment, the overall survival is still less than one year [61]. Rhabdomyosarcoma (RMS) is the most common childhood sarcoma. One of chemotherapeutics used in the treatment of RMS is CDDP [58]. Chemotherapy, including CDDP, is also a standard therapeutic regimen to treat Triple Negative Breast Cancer (TNBC) [62]. It is estimated that 50% of all cancer patients will be treated with CDDP and about a million of patients have received this drug in their anticancer therapies. 

Despite the fact that CDDP is a key chemotherapy drug in the treatment of patients with many types of cancer; serious side effects and MDR to this drug are very important clinical obstacles [3]. Therefore, there is an urgent need to look for novel therapeutic approaches including drugs and compounds from natural sources with weaker side effects. It has been demonstrated that natural compounds can sensitize to conventional cytotoxic therapy, intensify the drug effective concentration, reinforce the combined effect of agents or use cytotoxic effect specifically according to cancer cells. In addition, combined therapy using compounds which target multiple signaling pathways can reduce the development of MDR to anticancer drugs. The desired effect could be lower side effects of chemotherapeutic drug via replacing part of the dose of a traditional chemotherapeutic with a natural active agent with a defined effect. Numerous natural substances are well tolerated by cancer patients and do not cause serious toxic effects even at high doses. Interaction of conventional chemotherapeutic drugs with compounds of natural origin introduces a new aspect in the research and therapy of cancer [45]. Identification of a novel combination of chemotherapeutic strategy in which natural compounds can reduce the concentration of CDDP and minimize the adverse effects of conventional chemotherapy could work to good advantage for cancer patients.

With the development of comprehensive methods and techniques of plant metabolites’ isolation, identification, and bioactivity evaluation, new perspectives appeared in the case of MGN application. To the present, several mechanisms of action of MGN (individually or in combination) have been proposed. However, additional studies, including in vivo models on animals and on humans are needed to confirm the final biological activity, pharmacokinetics, and toxicity of MGN individually and in combination with CDDP. Moreover, the structural issues should be elucidated in the close future to exclude a potential presence of other chiral or stereoisomers of MGN in the natural sources. Other forms of MGN could have an important meaning in the toxicity studies and bioactivity assessment [21]. Side effects of application of MGN with CDDP also should be noted. More studies are needed to establish the best strategy to incorporate MGN into the CDDP-based therapy of patients with cancers, maximizing clinical benefits and minimizing toxicity.

## 5. Materials and Methods 

### 5.1. The Reagents

The reagents for extraction and chromatographic separation of MGN, namely methanol, methyl-*t*-butyl ether, hydrochloric acid and triethylamine were of reagent grade and were purchased from Avantor Performance Materials (Gliwice, Poland). HPLC analysis was performed using the chromatography grade acetonitrile, acetic acid (Merck, Darmstadt, Germany) and double-distilled water (Millipore, Temecula, CA, USA), whereas the mass spectrometry analysis of the isolate required the spectrometric purity water, acetonitrile, and formic acid, which were also delivered by Merck.

### 5.2. The Extraction and Chromatographic Analysis of MGN from Berberis siberica Overgound Parts

The overground parts of Siberian barberry (*Berberis siberica* Pall.) were collected from the Bayan province in Mongolia in September 2014 and were authenticated by Dr Daariimaa Khurelbat, the Head of the Department of Pharmaceutical Sciences and Pharmacognosy at Mongolian National University of Medical Sciences in Ulan-Bataar. A voucher specimen of the plant material is present in the Department of Pharmacognosy of Medical University of Lublin.

50 g of the dried and powdered overground parts of the plant were extracted by accelerated solvent extractor (ASE 100, Dionex, Sunnyvale, CA, USA) in the following conditions: extraction temperature of 100 °C, extraction time of 5 cycles 10 min each, purge time of 60 sec, flush volume of 60%. The pressure during the extraction process ranged around 95 bars. The obtained extracts were joined and evaporated to dryness at 45 °C, giving the final yield of the extract of 18%. 1 g of the obtained extract was dissolved in the 70:30 (v/v) mixture of the acidified lower phase and neutral upper phase and the separation was conducted on a hydrostatic CPC according to the previously described protocol, which used an ascending mode separation in a biphasic solvent system composed of water and methyl-*t*-butyl ether (1:1, v/v) with the addition of 10 mmol of hydrochloric acid to the stationary lower phase and trietylamine to the mobile upper phase, each. After an introduction of a stationary phase on the column, the extract was injected together with the mobile phase and was fractionated by an alkalified upper organic phase at 1050 rpm and a flow rate of 5 mL/min, as previously described [32]. The purity of the isolate was checked in an HPLC-MS analysis, where a gradient of acetonitrile (B) and water (A) with the addition of 0.1% of formic acid was used in the following way: 0 min—10% of B in A, 10 min—40% of B in A, 12 min—40% of B in A, and 17-20 min—95% of B in A, 22 min—10% of B in A, on a Zorbax RP 18 Stable Bond column (150 mm × 2.1 mm, d = 3.5 µm) (Agilent Technologies, Santa Clara, CA, USA). The method length was set at 30 min, the flow rate at 0.2 mL/min, the temperature at 25 °C, post-time at 5 min and the detection wavelength at 254, 280, 290, and 320 nm. The mass spectrometric analysis was performed using a 6500 Series ESI-Q-TOF-MS spectrometer produced by Agilent Technologies (Santa Clara, CA, USA) that is coupled with an LC system (1200 Series, Agilent Technologies, Santa Clara, CA, USA). The platform is composed of a degasser, a binary pump, an autosampler, a column thermostat, a PDA detector, and a spectrometer. The following operation conditions of mass spectrometer were applied: gas temperature: 350 °C, sheath gas flow: 325 °C, drying and sheath gas flows: 12 L min−1, nebulizer voltage: 30 psig, fragmentor voltage: 170 V, skimmer voltage: 65 V, capillary voltage: 4000 V, collision energies: 20 and 40V. All experiments were recorded in both positive and negative modes with an addition of internal standards (MW: 121.0508 and MW: 922.0097), in the *m*/*z* range of 100 to 1700 m/z. Mass Hunter Workstation software (version B.08.00) was used for the analysis of the recorded spectra.

### 5.3. Drug

Cisplatin (CDDP) was purchased from Sigma (St. Louis, MO, USA) and dissolved in phosphate buffered saline (PBS) with Mg^2+^ and Ca^2+^ at 1 mg/mL as a stock solution. The drug was diluted to obtain the final concentration with respective culture medium before use.

### 5.4. Cell Lines

TE671 human rhabdomyosarcoma (ATCC^®^ HTB-139™), T98G human glioblastoma (ATCC^®^ CRL-1690™), MDA-MB-468 human breast cancer (ATCC^®^ HTB-132™) and NCIH1299 non-small lung cancer (ATCC^®^ CRL-5803™) cells were obtained from the American Type Culture Collection (ATCC) (Manassas, VA, USA). TE671, T98G, and MDA-MB-468 cancer cell lines were grown in DMEM/F12 (Sigma, St. Louis, MO, USA), NCIH1299 cells were maintained in RPMI1640 (Sigma, St. Louis, MO, USA) culture medium supplemented with 10% FBS (Sigma, St. Louis, MO, USA), penicillin (Sigma, St. Louis, MO, USA) (100 IU/mL), and streptomycin (Sigma, St. Louis, MO, USA) (100 μg/mL). Mycoplasma-free cultures were kept in a humidified atmosphere of 95% air and 5% CO_2_ at 37 °C.

### 5.5. Cell Viability Assay

TE671, T98G, MDA-MB-468, and NCIH1299 cancer cells were placed on a 96-well plate (Nunc, Rochester, NY, USA) at a density of 1 × 10^4^ cells/mL. The next day, the culture medium was removed, and cells were exposed to serial dilutions of total extract of MGN (0.01–2 mg/mL) or CDDP (0.02–10 µg/mL) individually or in mixtures of both compounds for 96 h. Then, the cancer cells were incubated with the 3-(4,5-dimethylthiazol-2-yl)-2,5-diphenyltetrazolium bromide (MTT) solution at concentration 5 mg/mL (Sigma, St. Louis, MO, USA) for 3 h. During this time, MTT was metabolized by living cells to purple formazan crystals which were solubilized in a sodium dodecyl sulfate (SDS) buffer (10% SDS in 0.01 N HCl) overnight. The optical density of the product was measured at 570 nm using an Infinite M200 Pro microplate reader (Tecan, Männedorf, Switzerland). Dose–response curves were plotted to determine half-maximal inhibitory concentrations (IC_50_) for the CDDP and MGN using GraphPad Prism 5.0 (GraphPad Software, San Diego, CA, USA). The results of combined treatment MGN and CDDP were analyzed according to isobolographic protocol. The drug doses were determined based on the IC_50_ values calculated from the previous cytotoxicity test. 

### 5.6. Isobolographic Analysis of Interactions for Parallel and Nonparallel Concentration–Response Relationship Effects of MGN and CDDP

Pharmacodynamic nature of interactions between MGN and CDDP in four various cancer cell lines was analyzed by means of the type I isobolographic analysis for both parallel and nonparallel concentration–response relationship lines, as described previously [36,46]. To start the isobolographic analysis of interaction between MGN and CDDP, the inhibition of cell viability of TE671, T98G, NCIH1299, and MDA-MB-468 cancer cell lines was determined in the MTT test. Due to log-probit method, it was possible to determine median inhibitory concentrations (IC_50_ values) for MGN and CDDP in 4 cancer cell lines, as recommended elsewhere [39,49]. Log-probit analysis revealed that concentration–response relationship lines for MGN and CDDP were nonparallel to one another in the TE671, T98G, and MDA-MB-468 cancer cell lines. Simultaneously, the concentration–response relationship lines for MGN and CDDP were collateral in the NCIH1299 cancer cell line. This is the reason to use the type I isobolographic analysis for both nonparallel and parallel concentration–response relationship lines, as advised earlier [39,49,63,64]. To classify the type of interactions between MGN and CDDP, the comparison of the experimentally derived IC_50 exp_ values (at the fixed ratio of 1:1) with their respective theoretically calculated additive IC_50 add_ values was performed by means of the unpaired Student’s t-test with Welch’s correction, as recommended elsewhere [38,65,66]. The isobolographic analysis of interaction distinguishes four types of pharmacodynamic interaction: supra-additivity (synergy), additivity, sub-additivity (relative antagonism) and infra-additivity (absolute antagonism) [39,63,67].

### 5.7. Statistical Analysis

The IC_50_ and IC_50_ mix values for CDDP and MGN administered alone or in combination at the fixed ratio of 1:1 were calculated by computer-assisted log-probit analysis according to Litchfield and Wilcoxon [68]. The experimentally derived IC_50_ exp values for the mixture of CDDP with MGN were statistically compared with their respective theoretical additive IC_50_ add values by the use of unpaired Student’s t-test with Welch’s correction, according to Tallarida [66]. Results were analyzed using GraphPad Prism 5.0 software (one-way ANOVA; Tukey post-hoc testing). Statistical differences were considered relevant at *p* < 0.05 (* *p* < 0.05, ** *p* < 0.01, *** *p* < 0.001). Data are expressed as mean ± standard deviation of the mean (± SD).

## 6. Conclusions

In our manuscript we demonstrated that MGN strongly promoted CDDP-induced anti-proliferative effects in human breast, lung, rhabdomyosarcoma, or glioblastoma cancer cells. Moreover, MGN and CDDP applied together yielded synergistic or additive type of pharmacological interaction by means of isobolographic analysis. We hypothesize that combined treatment of CDDP with MGN, as a novel strategy for cancer treatment, would increase the anticancer effect of CDDP. However, further studies are needed to elucidate targets and molecular mechanisms of MGN/CDDP action. 

## Figures and Tables

**Table 1:** Half-maximal inhibitory concentrations (IC_50_) for the magnoflorine (MGN) and cisplatin (CDDP) in TE671, T98G, NCIH1299, and MDA-MB-468 cancer cells.

Cell Line	Drug	IC_50_ (μg/mL)	*n*	CFP	*q*/*p*	S.R.	f Ratio S.R.	Parallelism
TE671	MGN	22.83 ± 8.65	64	0.871 (*q*)	4.514	4.422	3.965	N.P.
TE671	CDDP	0.86 ± 0.49	80	0.193 (*p*)				
T98G	MGN	112.12 ± 48.06	80	0.518 (*q*)	0.310	5.664	2.251	N.P.
T98G	CDDP	3.18 ± 0.78	32	1.673 (*p*)				
NCIH1299	MGN	189.65 ± 48.97	48	1.246 (*q*)	2.898	1.513	1.860	P.
NCIH1299	CDDP	5.43 ± 1.86	48	0.430 (*p*)				
MDA-MB-468	MGN	187.32 ± 45.80	64	0.800 (*q*)	0.427	2.106	1.832	N.P.
MDA-MB-468	CDDP	1.91 ± 0.64	80	1.872 (*p*)				

Results are median inhibitory concentrations (IC_50_ values in μg/mL ± S.E.M.) of MGN and CDDP, when administered singly with respect to their anti-proliferative activity on four cancer cell lines (TE671, T98G, NCIH1299, and MDA-MB-468) measured in vitro by the MTT assay. *n*—total number of items used at concentrations of which expected anti-proliferative effects ranged between 4 and 6 probits (16% and 84%); CFP–(*q* and *p*) curve-fitting parameters; *q*/*p*—ratio of *q* and *p* values; S.R.—slope function ratio (S_CDDP_/S_MGN_); f ratio S.R.—factor for slope function ratio. Test for parallelism of two concentration–response relationship lines for MGN and CDDP was performed according to Litchfield and Wilcoxon [36]. If the slope function ratio (S.R.) value is higher than the factor for slope function ratio (f ratio S.R.) value, the examined two lines are nonparallel to each other [36]. Otherwise, if S.R. value is higher than f ratio S.R. value, the studied two lines are collateral each other [36]. N.P.—not parallel; P.—parallel. All detailed calculations necessary to confirm the parallelism of two concentration–response relationship lines of MGN and CDDP were presented in the Appendix to the paper by Luszczki and Czuczwar [37].

**Table 2:** Type I isobolographic analysis of interactions (for nonparallel and parallel concentration–response relationship lines) between magnoflorine (MGN) and cisplatin (CDDP) at the fixed drug concentration ratio of 1:1 in four cancer cell lines (TE671, T98G, NCIH1299, and MDA-MB-468).

Cell Line	IC_50 exp_ (μg/mL)	*n*_exp_	*L*-IC_50 add_ (μg/mL)	*n*_add_	*U*-IC_50 add_ (μg/mL)	*n*_add_	Interaction
TE671	13.80 ± 2.50	80	6.14 ± 5.23	140	17.55 ± 7.30	140	additivity
T98G	16.47 ± 4.84	80	35.37 ± 24.69	108	79.95 ± 27.00	108	additivity with tendency towards synergy
NCIH1299	36.53 ± 6.73 **	80	97.54 ± 20.02	92	-	-	synergy
MDA-MB-468	106.39 ± 36.24	80	67.46 ± 37.50	140	121.87 ± 44.06	140	additivity

Results are median inhibitory concentrations (IC_50_ values in μg/mL ± S.E.M.) for two-drug mixtures, determined either experimentally (IC_50 mix_) or theoretically calculated (IC_50 add_) from the equations of additivity [38], blocking proliferation in 50% of tested cells in four cancer cell lines (TE671, T98G, NCIH1299, and MDA-MB-468) measured in vitro by the MTT assay. *n*_exp_—total number of items used at those concentrations of which expected anti-proliferative effects ranged between 16% and 84% (i.e., 4 and 6 probits) for the experimental mixture; *n*_add_—total number of animals calculated for the additive mixture of the drugs examined (*n*_add_ = *n*__CDDP_ + *n*__MGN_ − 4); *L*-IC_50 add_ value calculated from the equation for the lower line of additivity; *U*-IC_50 add_ value calculated from the equation for the upper line of additivity. The unpaired Student’s *t*-test with Welch’s correction revealed that the IC_50 exp_ and IC_50 add_ values considerably differ indicating supra-additive (synergistic) interaction between MGN and CDDP in the NCIH1299 cancer cell line as measured by the MTT assay in vitro. ** *p* < 0.01 vs. the respective IC_50 add_ value.

## Abbreviations

ANOVA	Analysis of Variance
ATCC	American Type Culture Collection
CDDP	Cisplatin
CDK1	Cyclin-dependent Kinase 1
CDK2	Cyclin-dependent Kinase 2
CFP	Curve-fitting Parameters
CPC	Hydrostatic Countercurrent Chromatograph
CRAE	Coptidis Rhizome Aqueous Extract
DNA	Deoxyribonucleic Acid
DNA-Pt-HMGB1	Deoxyribonucleic Acid-Platinum-High Mobility Group Box 1
DOX	Doxorubicin
DRPs	DNA Damage Response Proteins
eEF2	Eukaryotic Elongation Factor 2
ESI-Q-TOF-MS	Electrospray Ionization–Quadrupole-Time-of-flight–Mass Spectrometry
f ratio S.R.	Factor for Slope Function Ratio
FBS	Fetal Bovine Serum
FDA	Food and Drug Administration
HCl	Hydrochloric Acid
HIV	Human Immunodeficiency Virus
HMGB1	High Mobility Group Box 1
HPLC-MS	High-performance Liquid Chromatography–Mass Spectrometry
IC_50_	The half Maximal Inhibitory Concentration
LC-MS	Liquid Chromatography–Mass Spectrometry
MDR	Multidrug Resistance
MGN	Magnoflorine
MS	Mass Spectrometry
MTT	3-(4,5-dimethylthiazol-2-yl)-2,5-diphenyltetrazolium bromide
N.P.	Not Parallel
P.	Parallel
p-AKT	Phospho-AKT
PBS	Phosphate-Buffered Saline
PDA	Photodiode Array
PI3K/PKB	Phosphatidylinositol-3-kinase/Protein Kinase B
p-mTOR	Phospho-mTOR
p-p38	Phospho-p38
RMS	Rhabdomyosarcoma
q/p	Ratio of q and p Values
S.E.M.	Standard Error of the Mean
S.R.	Factor for Slope Function Ratio
SD	Standard Deviation
SDS	Sodium Dodecyl Sulfate
VEGF	Vascular Endothelial Growth Factor
